# Frontier and hotspot evolution in Wiskott–Aldrich syndrome: A bibliometric analysis from 2001 to 2021

**DOI:** 10.1097/MD.0000000000032347

**Published:** 2022-12-16

**Authors:** Shixu Liu, Xiaoyan Yao, Kun Xia, Jinzhi Zhang, Yanyi Liu, Xiao Xia, Guangxi Li

**Affiliations:** a Guanganmen Hospital, China Academy of Chinese Medical Sciences, Beijing, China; b Graduate School of Beijing University of Chinese Medicine, Beijing, China.

**Keywords:** bibliometric analysis, CiteSpace, primary immunodeficiency, VOSviewer, Wiskott–Aldrich syndrome

## Abstract

Wiskott–Aldrich syndrome (WAS) is a rare X-linked primary immunodeficiency disorder. Despite our enormous progress in the strategies used to diagnose, treat, and cure WAS, no bibliometric studies have been performed in this research field. This study explored the trends in WAS research through a bibliometric analysis evaluating relevant literature quantitatively and qualitatively.

**Methods:**

The literature concerning WAS from 2001 to 2021 was retrieved from the Science Citation Index Expanded (SCI-expanded) of the Web of Science Core Collection database. Acquired data were then visually analyzed using CiteSpace and VOSviewer.

**Results:**

2036 papers were included in the final analysis. The annual publication outputs reached its peak in 2013 but declined in recent years. The dominant position of the United States in WAS research was quite obvious. Harvard University (USA), University College London (UK), and Inserm (France) were the three most prolific institutions. Adrian J. Thrasher exerted significant publication impact and made the most notable contributions in the field of WAS. *Blood* was the most influential journal with the highest publication outputs, and nearly all the top 10 journals and co-cited journals belonged to Q1. Immune dysregulation, thrombocytopenia, syndrome protein deficiency, stem cell, mutation, and diagnosis were the keywords with the strongest citation burst.

**Conclusion:**

From 2001 to 2021, the United States was a global leader in the WAS research. Collaboration between countries and institutions is expected to deepen and strengthen in the future. Research hotspots included pathogenesis, clinical manifestations, diagnosis, and therapy. Our results suggest a greater understanding of the mechanistic underpinnings of immune dysfunction in WAS patients, the application of targeted therapies for individual complications, and the development of curative approaches, which will remain research hotspots in the future.

## 1. Introduction

Wiskott–Aldrich syndrome (WAS, OMIM 301000) is a rare X-linked, complex primary immunodeficiency disease characterized by microthrombocytopenia (reduction in the number and size of platelets), eczema, recurrent infections, and increased risk of autoimmunity and malignancies.^[1,2]^ It was named after the two physicians who originally described the condition.^[3]^ In 1937, Alfred Wiskott, a German pediatrician, described three brothers who had frequent bleeding episodes, eczema, and recurrent ear infections since early infancy.^[4]^ Later, in 1954, Robert Aldrich, an American pediatrician, reported a similar clinical phenotype in sixteen out of forty males, but not females, over six generations of a single Dutch family, clearly demonstrating an X-linked mode of inheritance.^[5]^ The disease is also sometimes called the eczema-thrombocytopenia-immunodeficiency syndrome, keeping with the typical manifestation description.

Affecting between 1 in 50,000 and 1 in 250,000 live births,^[6]^ almost always boys only, WAS is caused by mutations in the *WAS* gene (ID: 7454) that impair or eliminate expression of the WAS protein (WASp).^[7]^ Over 400 pathogenic *WAS* variants spanning the gene sequence have been described^[3]^ and there is a genotype-phenotype correlation; the severity of the clinical phenotype can be predicted by the type of gene mutation and the latter’s impact on WASp expression levels.^[8]^ Mutations result in the absence of WASp cause the classic WAS phenotype. Mutations that result in reduced protein expression cause milder symptoms, usually referred to as X-linked thrombocytopenia (XLT). Affected individuals have small platelets with mild eczema and immunodeficiency, if any.^[9]^ Mutations in the GBD domain of WASp lead to X-linked neutropenia characterized by congenital neutropenia, myeloid dysplasia, and lymphoid cell abnormalities.^[10]^ Since X-linked neutropenia does not have the traditional WAS phenotype of thrombocytopenia and eczema, it should be differentiated from XLT and WAS. Patients with XLT may progress in severity, hence then, efforts have been launched to abandon the use of the XLT definition. Different clinical presentations are simply regarded as “mild” or “severe,” to raise awareness that even patients with milder types must be carefully monitored longitudinally.^[3]^

The diagnosis can be made on the basis of clinical features, blood smear, and abnormal immunoglobulin levels.^[11]^ The current gold standard for diagnosis is DNA sequence.^[12]^ Treatment of WAS depends on the severity of the disease. In cases of severe phenotype, the only curative approach currently available is hematopoietic stem cell transplantation.^[13,14]^ Otherwise treatment is focused on managing symptoms and preventing complications. Despite the description of WAS over 80 years ago, researchers and clinical workers are still making enormous progress in the understanding of the pathogenesis of WAS and its associated complications, as well as strategies used to manage the disorder.

Although these findings provide valuable insights and research directions for WAS in general, they can not provide a complete picture of global WAS research. For example, useful knowledge about developments in the domain, such as which are the most prolific countries, institutions, and journals; what national and institutional cooperation exist in the research; and what are the hotspots in the WAS research, are questions that difficult to answer utilizing traditional review methodologies. However, such information is helpful for researchers to understand the past evolution and the structure of the WAS research field and to explore the emerging trends of the domain. Hence then, we introduce bibliometric analysis for additional analysis of all scientific literature concerning WAS over the past two decades, which can provide additional insights and answer the questions above. It might be better than meta-analyses that do not highlight relationships between publications within the field or mere review articles that are not necessarily comprehensive enough.

Bibliometric analysis is a mathematical and statistical approach to exploring and analyzing research performance.^[15]^ Combining visualizing processing tools like CiteSpace^[16]^ and VOSviewer,^[17]^ it utilizes published data to identify novel findings and current research trends in specific fields. Therefore, clinical practitioners and researchers can update the new practices that evoke novel research ideas, providing the foundation for the subsequent studies.^[18,19]^ Over the years, bibliometric analysis has been applied to the medical field including rare diseases.^[20,21]^ To the best of our knowledge, bibliometric analyses on WAS are limited (none). This study aimed at exploring the hotspots and trends of this multifaceted and challenging disease by analyzing scientific literature from 2001 to 2021, to provide new visions for future researchers and clinical workers, especially for those who have curiosity but are novices in this field.

## 2. Materials and Methods

### 2.1. Data collection

All historic literature was culled from the Science Citation Index Expanded (SCI-expanded) of the Web of Science Core Collection database. According to the MeSH term, the search string was set to TS = (“Wiskott–Aldrich Syndrome” OR “eczema-thrombocytopenia-immunodeficiency syndrome”). The publication period was limited from 2001 to 2021. Of various document types, only articles and review articles written in English were included. Record content selected full record and cited references. Then exported the records as plain text files, and saved them in the format of download.txt. The entire process was conducted within one day (March 10, 2022) to reduce the bias conferred by database updating.

### 2.2. Data analysis

Bibliometric indicators involved the number of publications and citations for judging academic success. The number of publications (Np) is the yardstick of productivity, and the number of citations without self-citations (Nc) is used to measure the scientific impact. The *h*-index is a research-level metric that evaluates both the productivity and citation impact of the published papers by finding the threshold that connects Np with Nc. It is defined as the maximum value of *h* so that the given scholar has published at least *h* papers that have each been cited no less than *h* times.^[22]^ Although initially for the individual scholar, more recently *h*-index is applied to the productivity and impact of a country/region, an institution, or a journal.^[23]^ Moreover, the impact factor (IF) obtained from the latest version of Journal Citation Reports has been frequently used as a proxy for the relative importance of a journal within its field.^[24]^

Relevant data were imported into CiteSpace and VOSviewer to perform visual analysis. CiteSpace version 5.8.R3 (Drexel University, Philadelphia, PA) was applied to visualize the intercountry and institutional analysis and the collaboration between authors. Developed by Chen, Citespace is a tool for progressive knowledge domain visualization.^[25]^ It is particularly applicable for analyzing and visualizing patterns and trends in scientific literature. The main objective of knowledge domain visualization is to find key points in the development of research. CiteSpace gives a visual aid that characterizes the hotspots and evolution research processes and forecasts the future trends of the domain intuitively.^[16,26]^ VOSviewer version 1.6.17 (Leiden University, Leiden, Netherlands) was used to illustrate the references’ maps, as well as journal co-citation and keyword co-occurrence analysis. Unlike the conventional applications for constructing and viewing bibliometric networks, VOSviewer concentrates on the graphic representation of knowledge mappings. It is ideal for sizable bibliometrics display in an easy-to-explain way.^[17]^

## 3. Results

### 3.1. The trend of publication outputs

**Figure 1.**
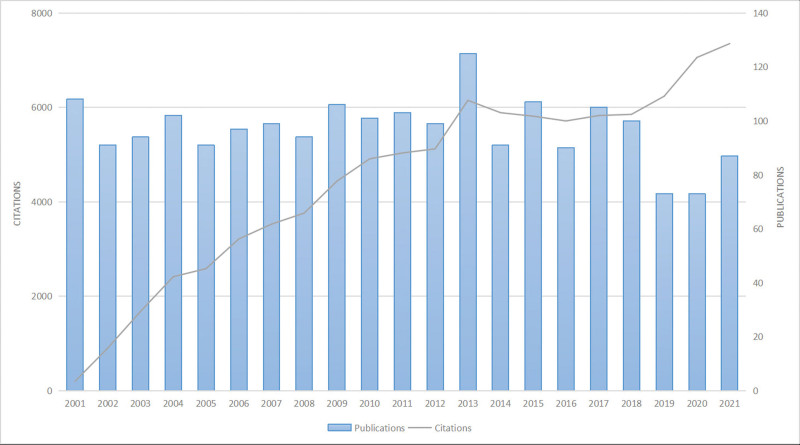
Trends of WAS publications over the past 20 years. WAS = Wiskott–Aldrich syndrome.

As shown in Figure 1, the number of published papers concerning WAS demonstrated an overall steady trend and reached its peak in 2013. A total of 2036 papers (including 1590 articles and 446 review articles) met the search strategy. The total citations without self-citations were 76,986, and the average number of citations was 46.42 per item. The *h*-index of all papers was 138. Although the publication outputs reduced in the past three years, the annual citations displayed a significant upward trend.

### 3.2. Contribution of countries/regions and institutions

**Figure 2.**
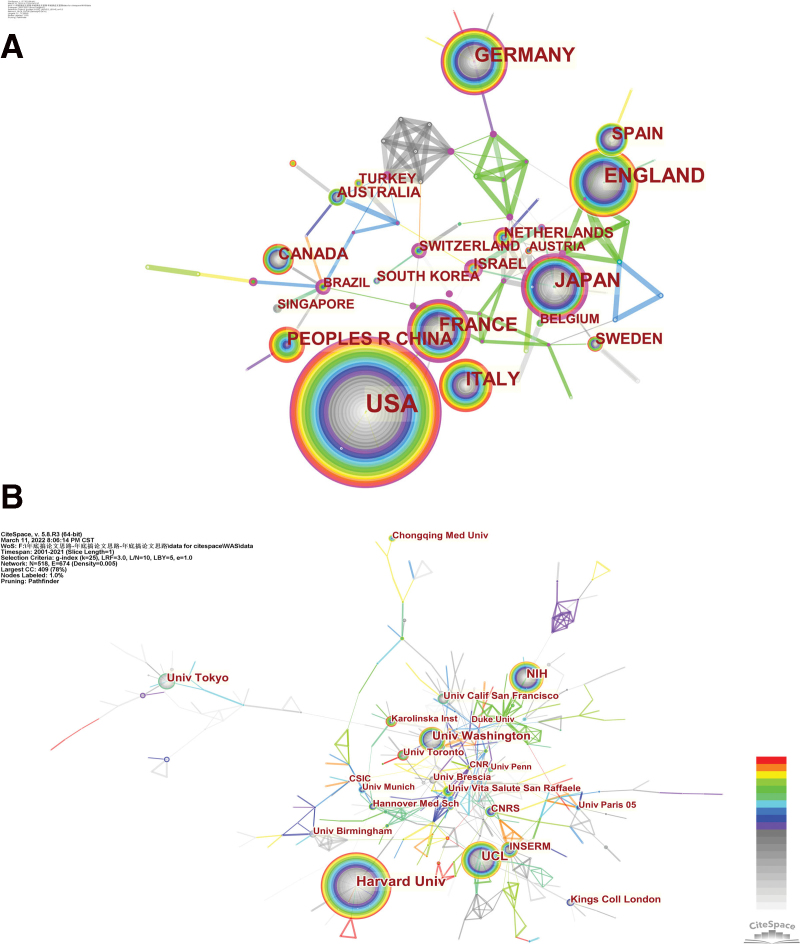
A. Distribution of publications from different countries/regions. B. Distribution of publications from different institutions. CNRS = French National Centre for Scientific Research (French Centre national de la recherche scientifique), INSERM = French National Institute of Health and Medical Research (French Institut national de la santé et de la recherche médicale), NIH = National Institutes of Health, UCL = University College London.

The maps of intercountry/regional (Fig. 2A) and inter-institutional cooperation (Fig. 2B) were visualized using CiteSpace. Tables 1 and 2 ranked the top 10 high-output countries/regions and institutions in this domain. The United States employed the highest number of papers (Np = 893, 43.86%), followed by the United Kingdom (Np = 256, 13.02%), Japan (Np = 209, 10.27%), Germany (Np = 207, 10.17%), and France (Np = 178, 8.74%). Articles from the USA had the highest citations (Nc = 49,505), accounting for 64.30% of the total, the UK also ranked second in this indicator (Nc = 14,469). Besides, the USA received the highest *h*-index (118), nearly twice the figure of the UK (70). Figure 1 also displayed the annual publications and growth trends of the top 10 countries/regions in WAS research. It is worth noting that despite China initially lagging behind, its annual publication outputs increased rapidly, outpacing the UK from 2020. Centrality measures the transformative potential of scientific contribution.^[27]^ Countries/regions such as Brazil, Ireland, and France had a high degree of centrality, as indicated by the node’s purple trims in Figure 2A. Such nodes tend to bridge different stages in the research development process.

**Table 1:** Top 10 countries/regions for publications and centrality in the WAS research (2001–2021).

No.	Country/region	Np (% of 2036)	Nc	*h*-index	Country/region	Centrality
1	USA	893 (43.86%)	49,505	118	Brazil	0.39
2	UK	265 (13.02%)	14,469	70	Ireland	0.3
3	Japan	209 (10.27%)	9493	50	France	0.28
4	Germany	207 (10.17%)	12,374	62	Israel	0.27
5	France	178 (8.74%)	9888	49	Russia	0.27
6	Italy	156 (7.66%)	8087	28	Serbia	0.27
7	China	121 (5.94%)	1783	23	Hungary	0.23
8	Spain	91 (4.47%)	2880	30	Finland	0.23
9	Canada	85 (4.17%)	5568	42	Czech Republic	0.22
10	Sweden	57 (2.80%)	2125	24	Switzerland	0.21

Nc = number of citations, Np = number of publications, WAS = Wiskott–Aldrich syndrome.

**Table 2:** Top 10 institutions for publications and centrality in the WAS research (2001–2021).

No.	Institutions	Country/region		Np	Nc	*h*-index	Institutions	Centrality
1	Harvard Univ		USA	189	11,271	61	CNRS	0.37
2	UCL		UK	155	8345	53	Univ Washington	0.29
3	Inserm		France	112	5432	42	Duke Univ	0.28
4	NIH		USA	107	4738	41	Leiden Univ	0.22
5	CNRS		France	80	3998	37	Burnham Inst Biomed Res	0.22
6	Univ Washington		USA	72	4516	37	Paris Descartes Univ	0.21
7	Univ Tokyo		Japan	66	4906	34	Natl Def Med Coll	0.21
8	Univ Toronto		Canada	50	3617	31	Tokyo Med and Dent Univ	0.2
9	Kings Coll London		UK	34	2082	27	Univ Brescia	0.16
10	Univ Calif San Francisco		USA	41	3369	31	INSERM	0.14

CNRS = French National Centre for Scientific Research (French Centre national de la recherche scientifique), Inserm = French National Institute of Health and Medical Research (French Institut national de la santé et de la recherche médicale), Nc = number of citations, NIH = National Institutes of Health, Np = number of publications, UCL = University College London, WAS = Wiskott–Aldrich syndrome.

Institutions with most highly publications, citations and *h*-index were Harvard University (Np = 189, Nc = 11,271, *h*-index = 61), followed by University College London (UCL, Np = 155, Nc = 8345, *h*-index = 53). The USA and the UK occupied the majority of positions on this list. In addition, French National Centre for Scientific Research (French: Centre national de la recherche scientifique, CNRS) had the highest centrality among all institutions. As shown in Figure 2A and B, There was some active cooperation among countries/regions and institutions, for instance, the USA, France, and Italy, UCL, and the French National Institute of Health and Medical Research (French: Institut national de la santé et de la recherche médicale, Inserm). Nevertheless, most countries/regions and institutions were dispersed and lack intensive collaboration.

### 3.3. Authors and co-cited authors

**Figure 3.**
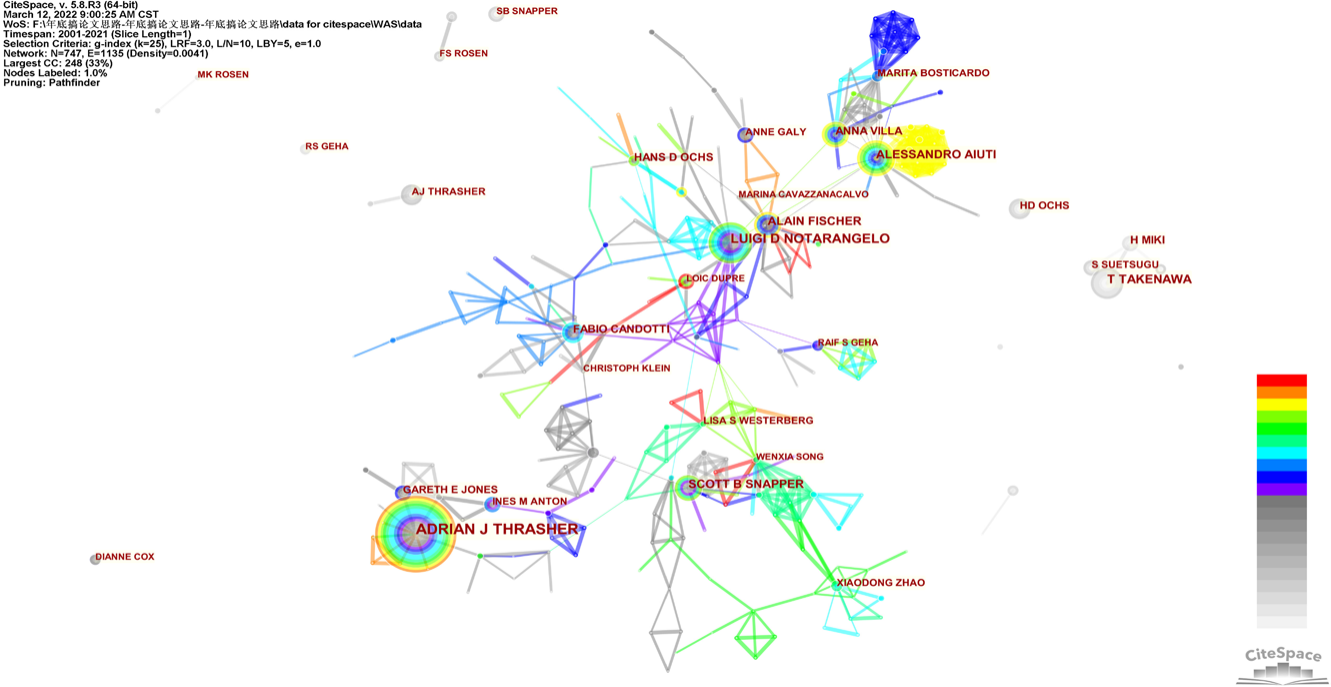
The active authors in the WAS research fields. WAS = Wiskott–Aldrich syndrome.

Table 3 listed the top 10 productive authors, highly cited authors and co-cited authors. Adrian J. Thrasher was the most prolific and highly cited scholar (Np = 90, Nc = 4496, *h*-index = 41), followed by Luigi D. Notarangelo (Np = 52, Nc = 1212, *h*-index = 30), Tadaorni Takenawa (Np = 45, Nc = 3336, *h*-index = 32), and Hans D. Ochs (Np = 45, Nc = 1368, *h*-index = 32). Figure 3 generated by CiteSpace demonstrated that there was an obvious cooperation network between different authors, such as Adrian J. Thrasher, Ines M. Anton, and Gareth E. Jones. Adrian J. Thrasher also had the highest degree of centrality (0.1) among all scholars. Generally, centrality ≥ 0.1 is regarded as relatively essential nodes, which implies that scholars significantly impact other’s work and studies from other groups.

**Table 3:** Top 10 authors, cited authors, and co-cited authors in the WAS research (2001–2021).

No.	Author	Np	*h*-index	Centrality	Cited author	Nc	Co-cited author	Nc
1	AJ Thrasher	90	41	0.10	AJ Thrasher	4496	H Miki	688
2	LD Notarangelo	52	30	0.04	T Takenawa	3336	HD Ochs	567
3	T Takenawa	45	32	0.01	H Miki	2089	SB Snapper	481
4	HD Ochs	45	32	0.03	HD Ochs	1368	R Rohatgi	480
5	SB Snapper	40	26	0.05	A Aiuti	1317	LM Machesky,	454
6	A Aiuti	37	25	0.03	LD Notarangelo	1212	JMJ Derry	403
7	F Candotti	34	23	0.03	HD Ochs	1188	HN Higgs	380
8	A Fischer	33	22	0.01	S Suetsugu	1092	AJ Thrasher	365
9	RS Geha	31	22	0.02	SB Snapper	1078	S Suetsugu	355
10	GE Jones	30	22	0.01	A Villa	948	S Linder	321

Nc = number of citations, Np = number of publications, WAS = Wiskott–Aldrich syndrome.

Two authors are considered as being co-cited when one or more article from each author’s oeuvre occurs in the same reference list.^[28]^ Among all the co-cited authors, six of them had a citation frequency of more than 400 times. Hiroaki Miki (688) was the most frequently cited author, followed by Hans D. Ochs (567).

### 3.4. Journals and co-cited journals

578 academic journals were involved in WAS research domain. *Blood* contributed the majority of published papers with the highest citations and *h*-index (Np = 100, Nc = 6209, *h*-index = 48). Moreover, it had the highest impact factor among the top 10 journals, followed by *Journal of Biological Chemistry* (Np = 77, Nc = 3836, *h*-index = 39).

The number of times that a scientific journal is co-cited, is an important metric to measure whether the journal has a considerable impact within a field. Five journals had been co-cited more than 4000 times among the top 10 co-cited journals. Again, *Blood* and *Journal of Biological Chemistry* came in first and second place in co-citations respectively. As shown in Table 4, nearly all the top 10 journals and co-cited journals belong to Q1 or Q2 based on the latest Journal Citation Reports in 2020.

**Table 4:** Top 10 journals and co-cited journals in the WAS research (2001–2021).

No.	Journal	Np	*h*-index	Nc	IF	JCR	Journal	Nc	IF	JCR
1	Blood	100	48	6209	22.113	Q1	Blood	10,104	22.113	Q1
2	J Biol Chem	77	39	3836	5.157	Q2	J Biol Chem	5104	5.157	Q2
3	J Allergy Clin Immunol	45	25	2110	10.793	Q1	Proc Natl Acad Sci USA	4212	11.205	Q1
4	Proc Natl Acad Sci USA	44	33	3336	11.205	Q1	J Cell Biol	4026	10.539	Q1
5	J Cell Sci	41	29	3259	5.285	Q2	Nature	3590	49.962	Q1
6	J Immunol	33	25	1659	5.422	Q2	J Immunol	3548	5.422	Q2
7	Front Immunol	28	11	373	7.561	Q2	Cell	3478	41.584	Q1
8	J Cell Biol	26	25	2287	10.539	Q1	Science	3002	47.728	Q1
9	J Clin Immunol	25	14	563	8.317	Q1	J Exp Med	2662	14.307	Q1
10	Mol Biol Cell	25	19	1598	4.138	Q3	J Allergy Clin Immunol	2487	10.793	Q1

JCR = Journal Citation Reports, Nc = number of citations, Np = number of publications, WAS = Wiskott–Aldrich syndrome.

### 3.5. Co-cited references and references burst

**Figure 4.**
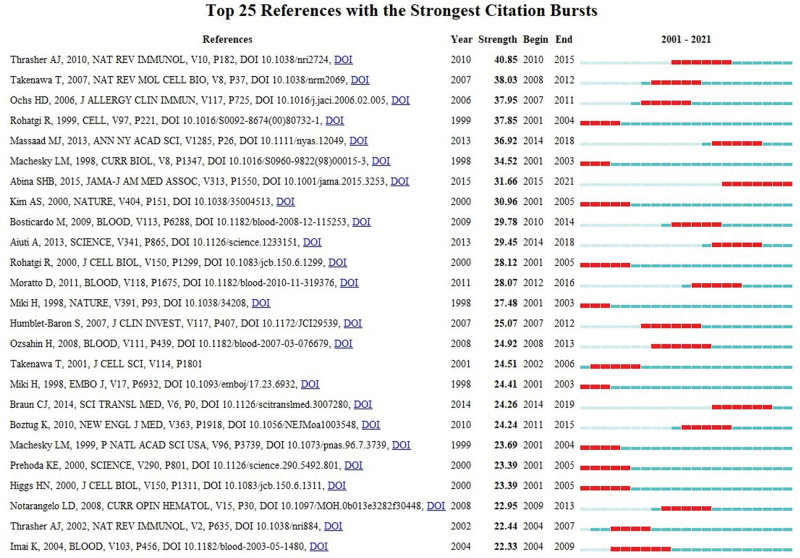
25 references with the strongest citation bursts related to WAS. WAS = Wiskott–Aldrich syndrome.

Co-citation is defined as the frequency that two or more references are cited simultaneously by at least one later publication. The more co-citations two references received, the higher their co-citation strength, and the more likely they are semantically related.^[29]^ Table 5 presented the 10 most often co-cited references among the retrieved references. Article titled *Isolation of a novel gene mutated in Wiskott–Aldrich syndrome* published in *Cell* by JM Derry in 1994 ranked the first.^[7]^ Citation burst provides evidence that a particular publication is associated with a surge of citations. Generally, articles with strong citation bursts are paid more attention by scholars in this research domain. Figure 4 generated by CiteSpace discloses that the first burst of citation began in 2001. The article published in *JAMA* by SHB Abina in 2015 was the most recent active citation burst.^[30]^ Most documents on the list had been cited constantly in the past twenty years. We can infer that the research concerning WAS may continue to prosper in the future.

**Table 5:** The top 10 co-cited references in the WAS research (2001–2021).

No.	References	Author	Year	Journal	Citation
1	Isolation of a novel gene mutated in Wiskott–Aldrich syndrome	JM Derry	1994	Cell	338
2	A multiinstitutional survey of the Wiskott–Aldrich syndrome	KE Sullivan	1994	J Pediatr	253
3	Wiskott–Aldrich syndrome protein-deficient mice reveal a role for WASP in T but not B cell activation	SB Snapper	1998	Immunity	238
4	Autoinhibition and activation mechanisms of the Wiskott–Aldrich syndrome protein	AS Kim	2000	Nature	236
5	The interaction between N-WASP and the Arp2/3 complex links Cdc42-dependent signals to actin assembly	R Rohatgi	1999	Cell	235
6	Wiskott–Aldrich syndrome protein, a novel effector for the GTPase CDC42Hs, is implicated in actin polymerization	M Symons	1996	Cell	210
7	Scar1 and the related Wiskott–Aldrich syndrome protein, WASP, regulate the actin cytoskeleton through the Arp2/3 complex	LM Machesky	1998	Curr Biol	190
8	Antigen receptor–induced activation and cytoskeletal rearrangement are impaired in Wiskott–Aldrich syndrome protein–deficient lymphocytes	J Zhang	1999	J Exp Med	187
9	N‐WASP, a novel actin‐depolymerizing protein, regulates the cortical cytoskeletal rearrangement in a PIP2‐dependent manner downstream of tyrosine kinases	H Miki	1996	EMBO J	184
10	Clinical course of patients with WASP gene mutations	K Imai	2004	Blood	179

WAS = Wiskott–Aldrich syndrome, WASp = WAS protein.

### 3.6. The analysis of hotspots and frontiers

Keyword co-occurrence analysis offers assistance to identify knowledge evolution and publication trends in a specific research field.^[25]^ According to Table 6, the top 10 keywords with the highest frequency in 2001–2021 were arp2/3 complex (413), n-wasp (369), activation (303), wasp (283), syndrome protein (215), actin polymerization (170), expression (167), actin (160), cytoskeleton (149), mutations (137).

**Table 6:** Top 20 keywords in the WAS research (2001–2021).

Rank	Keywords	Count	Rank	Keywords	Count
1	wiskott–aldrich-syndrome	681	11	mutations	137
2	arp2/3 complex	413	12	actin cytoskeleton	135
3	n-wasp	369	13	chronic granulomatous-disease	127
4	activation	303	14	stem-cell transplantation	122
5	wasp	283	15	severe combined immunodeficiency	116
6	syndrome protein	215	16	bone-marrow-transplantation	109
7	actin polymerization	170	17	t-cells	108
8	expression	167	18	immunological synapse	102
9	actin	160	19	cdc42	100
10	cytoskeleton	149	20	immunodeficiency	100

WAS = Wiskott–Aldrich syndrome, WASp = WAS protein.

**Figure 5.**
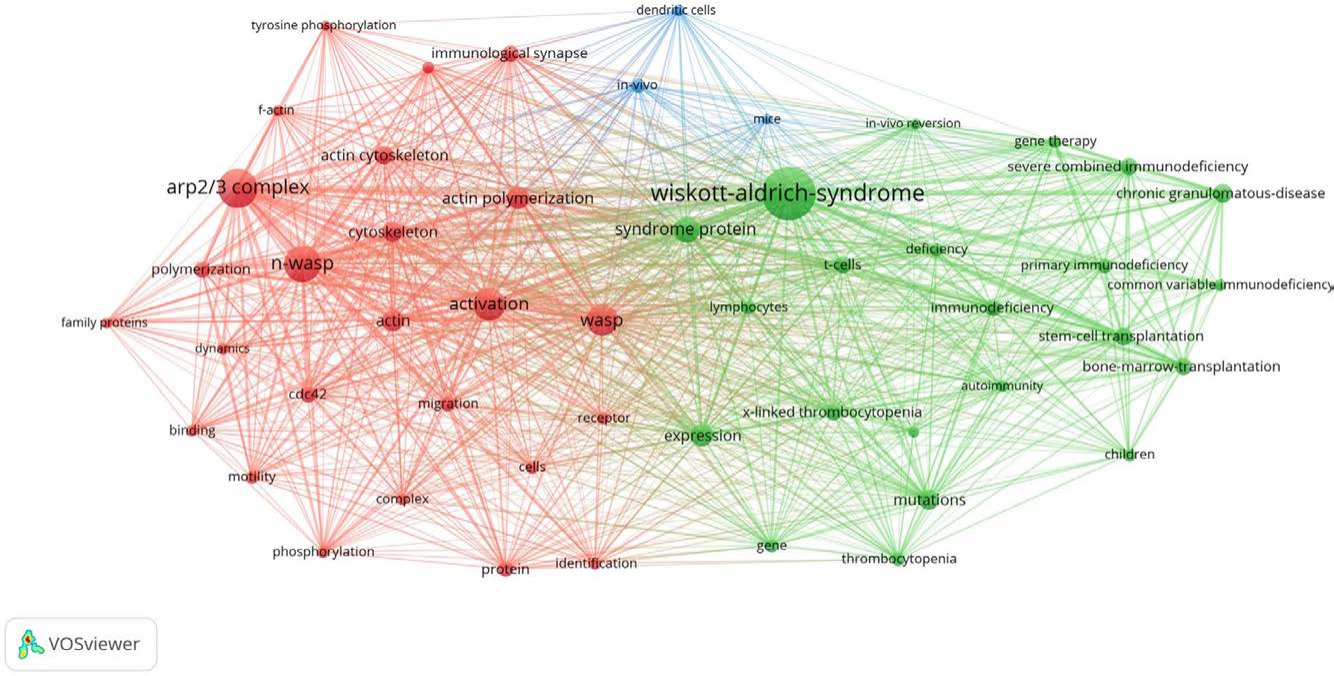
Keywords clustering analysis of the WAS research. WAS = Wiskott–Aldrich syndrome.

Keyword Clustering is combining similar, relevant queries into groups and using whole groups instead of separate keywords, which helps reflect knowledge core structure. We use VOSviewer for this procedure. Nodes and labels constitute a unit, and units with different colors build different clusters. There were red, green, and blue clusters as shown in Figure 5, which represented three research directions respectively. The main keywords of the red cluster were actin, actin cytoskeleton, activation, arp2/3 complex, binding, cdc42, cell, dynamics, family proteins, identification, immunological synapse, migration, motility, n-wasp, phosphorylation, polymerization, receptor, wip. The green cluster covered autoimmunity, bone marrow transplantation, children, chronic granulomatous disease, common variable immunodeficiency, deficiency, disease, expression, gene, gene therapy, immunodeficiency, in vivo reversion, lymphocytes, mutations, stem-cell transplantation, syndrome protein, thrombocytopenia. The blue cluster mainly included dendritic cells, in vivo, and mice.

Strong citation bursts can capture burst keywords and show cutting-edge topics over time. Table 7 listed the top 20 keywords with the strongest citation bursts and sorted them by their strength. The top five hotspots were: mutation (12.53), binding protein (10.82), chronic granulomatous disease (9.53), deficiency (9.34), and stem cell transplantation (9.19). Besides, keywords such as “children,” “mutation,” “thrombocytopenia,” “immunodeficiency,” “diagnosis,” “management,” “stem cell,” and “hematopoietic cell transplantation,” turned up constantly during the past five years (Table 8).

**Table 7:** Top 20 keywords with the strongest citation bursts in the WAS research (2001–2021). The red bars indicate the duration of the citation burst, whereas the blue bars represent the time interval.

Keywords	Strength	Begin	End	2001–2021
mutation	12.53	2018	2021	
binding protein	10.82	2002	2004	
chronic granulomatous disease	9.53	2014	2021	
deficiency	9.34	2015	2021	
stem cell transplantation	9.19	2014	2017	
listeria monocytogene	9.16	2001	2004	
filopodium formation	7.82	2001	2007	
thrombocytopenia	7.49	2017	2021	
polymerization	7.42	2001	2007	
actin depolymerizing protein	7.29	2001	2004	
mice	7.29	2012	2018	
depolymerizing protein	7.23	2001	2005	
homeostasis	7.05	2010	2015	
metastasis	6.79	2014	2017	
children	6.48	2016	2021	
phosphatidylinositol 4	6.47	2002	2005	
immunodeficiency	6.37	2017	2019	
rac	6.35	2001	2007	
lamelipodia	6.27	2005	2007	
b cell	6.21	2015	2019	

WAS = Wiskott–Aldrich syndrome.

**Table 8:** The strongest citation bursts keywords concerning WAS research after 2016.

Keywords	Strength	Begin	End	2016–2021
children	6.48	2016	2021	
reconstitution	5.86	2016	2019	
invasion	5.15	2016	2021	
immune dysregulation	5.08	2016	2018	
managment	4.68	2016	2021	
thrombocytopenia	7.49	2017	2021	
immunodeficiency	6.37	2017	2019	
outcm	6.07	2017	2021	
syndrome protein deficiency	5.94	2017	2021	
stem cell	5.48	2017	2021	
mutation	12.53	2018	2021	
diagnosis	5.26	2018	2021	
hematopoietic cell transplantation	3.6	2019	2021	
protein	5.71	2019	2021	

WAS = Wiskott–Aldrich syndrome.

## 4. Discussion

In this study, we conducted a bibliometric analysis to explore the developmental trends and hotspots of research on WAS from the SCI-expanded database by using CiteSpace and VOSviewer. We retrieved 2036 original articles and reviews published from 2001 to 2021. The annual publications demonstrated an overall steady trend (average 90 papers per year) and rebounded in 2021. Moreover, the annual citations were continuously on the rise, suggesting that the research on WAS is becoming more mature and has attracted the attention of more scholars.

From the perspective of countries and institutions, we can see that the United States, the United Kingdom, Japan, Germany, and France were the leading countries in WAS research. Except for the University of Toronto (Canada), all the top 10 productive institutions were from these countries. Harvard University was the biggest producer of high-quality WAS research. Centrality measures the value of a node that acts as a bridge in the network structure. Countries or institutions with high centrality (≥0.1) mean that they played important bridging roles in the global cooperation network. However, as shown in Figures 2A, B, and 3, The cooperation network is not well established, and the breadth and intensity of cooperation were not ideal. For instance, the USA and the UK, two research powerhouses, had less academic collaboration; Most collaborating institutions were limited to domestic ones. Considering its rarity, this situation may hinder the development of WAS research. It is highly recommended that research centers worldwide should remove barriers and promote collaboration to boost the development of WAS research.

As for authors, Adrian J. Thrasher was the most prolific scholar with 90 published papers. Besides, Thrasher had the highest citations (4496) and *h*-index (41), which reveals the most significant contributions he had made for WAS during the last two decades. Adrian Thrasher, a British immunologist, is Professor of Paediatric Immunology at the Institute of Child Health UCL. His research interests fall into two broad categories: the pathophysiology of primary immunodeficiency diseases and the development of somatic gene therapy. Professor Thrasher has played a leading role in defining the basic cytoskeletal defect in hematopoietic cells in WAS.^[31]^ He and his team have conducted some of the first successful trials of somatic gene therapy for primary immunodeficiency diseases including WAS.^[32]^

As shown in the analysis of journals*, Blood* and *Journal of Biological Chemistry* were the two most productive and influential journals. Nearly all the journals in Table 4 had a high impact factor and belong to Q1. This means that publishing original research on WAS in high-quality journals is not a challenge. Source literature analysis helps to discover the core journals within the research field. It was observed that cited articles are all from journals with high impact factors, implying that these journals had published a greater amount of breakthroughs within this field, which reminded scholars interested in this topic to pay more attention to these sources.

Table 5 showed the most frequently co-cited article was published by JM Derry et al in *Cell* in 1994, which pioneeringly isolated a novel gene, *WAS* from the X chromosome (position Xp11.22–p11.23). This is of historical interest and captures the initial description of the *WAS* gene, which considerably changed our concepts of WAS pathogenesis. In the same year, KE Sullivan et.al. conducted a multi-institutional retrospective survey to collect basic clinical and laboratory information on patients with WAS. The most recent co-cited article was published in 2004 by K Imai, which reported a strong phenotype-genotype correlation in patients with *WAS* mutation with more detailed observations.

Keyword co-occurrence analysis (Table 6) and keyword clustering analysis (Fig. 5) determine the research hotspots and frontiers. Our results indicated that the understanding of the pathophysiological underpinnings of this disorder, and the role of actin assembly and disassembly as they correlate to WAS was always the hot focus of research. In brief, the gene responsible for WAS is comprised of 12 exons and encodes a 502-amino acid cytosolic protein called WAS protein (WASp),^[7]^ which is involved in adaptive and innate immunity via regulation of actin cytoskeleton-dependent cellular processes. Deficiency in WASp connects perturbations in actin assembly with T cell, B cell, and myeloid cell abnormalities,^[33]^ as well as associated complications including infection, autoimmunity, inflammation, and malignancy.^[11]^ Subsequently, keywords such as “mutation,” “thrombocytopenia,” “immunodeficiency,” “immune dysregulation,” “stem cell,” and “hematopoietic cell transplantation” appeared frequently in the last 5 years, indicating that the mechanistic underpinnings of immune dysfunction and treatment modalities will remain research hotspots over the next few years. Our analysis also suggests that scholars are still trying to develop a better understanding of WAS. Basic and clinical research efforts across a wide array of disciplines, including immunology, hematology, and cellular therapy, continue to ensure a bright future for WAS patients.

However, there were some limitations in our study. Firstly, the database is updated continuously and dynamically. Hence, our results are essentially temporary and by default will not incorporate ongoing and future research areas. Secondly, only English publications from SCI-expanded databases were included. Therefore, a discrepancy may exist between our analysis and the real publication characteristics.

## 5. Conclusions

This bibliometric analysis reveals that research on WAS continued to make advances in the past two decades. The USA and UK had made many outstanding breakthroughs in this field. The vast majority of articles concerning WAS are published and cited in influential journals. Among them, *Blood* and *Journal of Biological Chemistry* were the two most representative ones. The hotspots over the decades were pathophysiology, clinical features, diagnosis, and therapies. Our results indicate that the mechanisms of WAS, novel approaches to current treatment, and other emerging therapies will remain research hotspots in the future.

## Acknowledgments

The authors thank Guang’anmen Hospital, China Academy of Chinese Medical Sciences, for their support of this work and the reviewers for allowing us to make improvements to the manuscript.

## Author contributions

SXL and GXL conceived the study. SXL, XYY, and KX collected the data. KX, JZZ, and YYL reexamined the data. SXL, XYY, and XX analyzed the data. SXL wrote the manuscript. GXL reviewed the manuscript. YYL and XX revised the manuscript. All authors contributed to the article and approved the submitted version.

**Conceptualization:** Shixu Liu, Guangxi Li.

**Data curation:** Xiaoyan Yao, Xia Kun, Jinzhi Zhang, Xiao Xia.

**Formal analysis:** Shixu Liu, Xiaoyan Yao.

**Funding acquisition:** Guangxi Li.

**Methodology:** Shixu Liu.

**Project administration:** Shixu Liu.

**Resources:** Shixu Liu.

**Software:** Shixu Liu.

**Validation:** Shixu Liu, Xia Kun, Yanyi Liu.

**Visualization:** Shixu Liu, Xiao Xia.

**Writing – original draft:** Shixu Liu.

**Writing – review & editing:** Guangxi Li.
